# SUMOylation mediates the disassembly of the Smad4 nuclear export complex via RanGAP1 in KELOIDS

**DOI:** 10.1111/jcmm.17216

**Published:** 2023-03-14

**Authors:** Xiaohu Lin, Qianqian Pang, Jie Hu, Jiaqi Sun, Siya Dai, Yijia Yu, Jinghong Xu

**Affiliations:** ^1^ Department of Plastic and Reconstructive Surgery Zhejiang Provincial People's Hospital People's Hospital of Hangzhou Medical College Hangzhou China; ^2^ Ningbo Hwamei Hospital University of Chinese Academy of Sciences, Ningbo Zhejiang China; ^3^ Department of Plastic Surgery The First Affiliated Hospital, School of Medicine Zhejiang University Hangzhou China

**Keywords:** CRM1, nuclear export, nucleocytoplasmic transport, RanGAP1*SUMO1, Smad4, sumoylation

## Abstract

Sentrin/small ubiquitin‐like modifier (SUMO) has emerged as a powerful mediator regulating biological processes and participating in pathophysiological processes that cause human diseases, such as cancer, myocardial fibrosis and neurological disorders. Sumoylation has been shown to play a positive regulatory role in keloids. However, the sumoylation mechanism in keloids remains understudied. We proposed that sumoylation regulates keloids via a complex. RanGAP1 acted as a synergistic, functional partner of SUMOs in keloids. Nuclear accumulation of Smad4, a TGF‐β/Smad pathway member, was associated with RanGAP1 after SUMO1 inhibition. RanGAP1*SUMO1 mediated the nuclear accumulation of Smad4 due to its impact on nuclear export and reduction in the dissociation of Smad4 and CRM1. We clarified a novel mechanism of positive regulation of sumoylation in keloids and demonstrated the function of sumoylation in Smad4 nuclear export. The NPC‐associated RanGAP1*SUMO1 complex functions as a disassembly machine for the export receptor CRM1 and Smad4. Our research provides new perspectives for the mechanisms of keloids and nucleocytoplasmic transport.

## 1 INTRODUCTION

Posttranslational modifications (PTMs) play essential roles in the regulation of biological processes. The rapid and reversible characteristics of PTMs contribute long‐term effective maintenance of their regulation.^1^, ^2^ Sumoylation has been widely studied as a PTM in recent years. More than 3000 substrate proteins have been found to be sumoylated under certain conditions, most of which are nucleoproteins.^3^, ^4^ According to the characteristics of the modified proteins, the biological functions of sumoylation include the regulation of subcellular localization, protein stability, and transcriptional activity and the maintenance of nucleolar and chromatin structures.^4^, ^5^, ^6^, ^7^, ^8^, ^9^ Five sentrin/small ubiquitin‐like modifiers (SUMOs) have been found in mammals, among which SUMO1, SUMO2 and SUMO3 are the most widely studied.^10^, ^11^ Sumoylation is also a dynamic and reversible process, that is catalysed by E1‐activating enzymes, E2‐conjugating enzymes and E3 ligases and SUMO moieties can be deconjugated from target proteins by sentrin/SUMO‐specific proteases (SENPs).^5^, ^12^

An increasing number of diseases for which sumoylation is involved in the occurrence and development have been reported. For example, sumoylation plays an important role in the epithelial inflammatory signal of inflammatory bowel disease. It has also been reported that sumoylation is involved in the development of myocardial fibrosis and liver fibrosis.^13^, ^14^, ^15^ The pathological nature of keloids entails excessive fibrosis of wounds.^16^ Studies have shown that sumoylation plays a positive regulatory role in keloids. However, in our previous study, when we knocked down SUMO proteases in keloids, the downstream phenotype of keloids was not consistent with the content of free SUMOs.^17^ Cheng et al.^18^ also found a controversial effect of sumoylation on the stability of substrate proteins compared with that reported in previous studies in mice lacking proteases. Therefore, we speculate that the regulation of sumoylation in keloids may be mediated by some specific SUMO complexes.

Ran is a nuclear Ras‐like GTPase that has two forms: one bound with GTP and one bound with GDP. Similar to other GTPases, Ran is said to function as a molecular switch involved in nucleocytoplasmic transport by associating with and dissociating from interacting proteins through conformational changes induced by nucleotide exchange or GTP hydrolysis.^1^, ^19^ Nucleotide exchange of Ran is catalysed by the GTP exchange factor RCC1, while GTP hydrolysis is catalysed by the GTPase‐activating protein RanGAP1.^1^ RanGAP1 was the first identified substrate for modification by SUMO1. There are two forms of RanGAP1: one is in the cytoplasm, and the other is covalently modified by SUMOs and is concentrated at the cytoplasmic fibres of nuclear pore complexes (NPCs).^20^ Unlike other sumoylated targets, for which only a minor fraction is conjugated at any one time, the majority of the RanGAP1 content is sumoylated. SUMO conjugation localizes RanGAP1 to the cytoplasmic face of NPCs to form stable tetramer complexes with RanBP2 and Ubc9 to protect them from the deconjugation activities of SUMO proteases.^21^ The RanBP2/RanGAP1*SUMO1/Ubc9 complex is a functional E3 ligase on a physiologically relevant target, which is involved in mitosis and nucleocytoplasmic transport, but its specific role remains rarely studied.^22^, ^23^

In this study, we aimed to explore the regulatory mechanism of sumoylation in keloids. As an intermediate carrier of sumoylation, RanGAP1*SUMO1 is hypothesized to mediate the nucleocytoplasmic transport of proteins in related signalling pathways and regulate the spatial distribution of intracellular proteins, affecting the downstream phenotype of keloids. The results of these study provide novel ideas for the understanding of sumoylation and nucleocytoplasmic transport, directions for relevant research and clues for the clinical treatment and drug development.

## 2 MATERIALS AND METHODS

### 2.1 Patients and specimens

Human keloid specimens were obtained from the patients received keloid excision surgery from 2019 to 2021 at the Department of Plastic Surgery, the First Affiliated Hospital of Zhejiang University. Written informed consents were obtained from each patient prior to surgery. All procedures in this study were approved by the hospital ethics committee. Detailed information about these patients is presented in Table S1.

### 2.2 Cell culture

Human keloid fibroblasts (HKFs) were isolated from keloid specimens. Tissues were digested by collagenase type I (Solarbio) and trypsin (Gibco). HKFs at passages four to six were used in the present studies. All cells were routinely cultured in Dulbecco's modified Eagle's medium (DMEM, BI) supplemented with 10% foetal bovine serum (FBS, Gibco), 100 U/ml penicillin (Gibco) and 100 mg/ml streptomycin (Gibco). Cells were incubated in 5% CO2, 37°C incubator (Thermo Scientific) with a humidified atmosphere.

### 2.3 Bioinformation databases

Publicly available datasets were analysed in this study. The PPI network was predicted using STRING database (http://www.string‐db.org/). STRING provided insights into the proteins associated with SUMOs. The interaction with top10 score was showed. The correlation between the SUMOs and RanGAP1 was from cBioPortal database (http://www.cbioportal.org/). A total of 124 samples of skin‐related carcinoma with data available in both axes were analysed according to mRNA expression. The expression of RanGAP1 in various organs was downloaded from the Human Protein Atlas (HPA, http://www.proteinatlas.org/).

### 2.4 Immunofluorescence

Human keloid fibroblasts were fixed with 4% paraformaldehyde solution (Servicebio) for 15 min and permeabilized in 0.1% Triton X‐100 for 20 min on sterile glass slips in six‐well plates. The slips were blocked with 5% bovine serum albumin (BSA, BBI) and incubated with primary antibody at 4°C overnight. Secondary antibody was used to incubate the cells for 1 h in the dark. Nuclei were stained with DAPI (Servicebio). The images were obtained by a fluorescence microscope (Olympus) and confocal microscopy. Quantitative analysis of fluorescence intensity was used with Image J. All the antibody employed in this study are listed in Table S2.

### 2.5 RNA extraction and RT‐PCR

Total RNA was extracted using RNA‐Quick Purification Kit (YiShan Biotech). The cDNA was reverse transcribed using HiScript II QRT SuperMix for qPCR (Vazyme Biotech). QRT‐PCR was performed with a CFX96TM Real‐Time System (BioRad) using SYBR^®^ Premix Ex Taq™ II (Takara) according to the manufacturer's instructions. The relative RNA amount was calculated by 2^−ΔΔCt^ method and normalized to the level of GAPDH. All primers used in this study are listed in Table S3.

### 2.6 Protein extraction and Western blotting

Total protein was extracted using RIPA lysis buffer (Beyotime) supplemented with protease and phosphatase inhibitor cocktails (Thermo Scientific). Nuclear and cytoplasmic protein was extracted, respectively, using NE‐PER Nuclear and Cytoplasmic Extraction Reagents (Thermo Scientific). A Bicinchoninic Acid Protein Assay Kit (Thermo Scientific) was used to quantify the protein content. The equal amount of protein was separated by 4%–12% SDS‐PAGE (GenScript) and transferred to 0.22 μm PVDF membranes (Millipore). The membranes were blocked with 5% non‐fat milk in TBST for 1 h and incubated with primary antibody at 4°C overnight. After washing three times with TBST, the membranes were incubated with HRP‐conjugated secondary antibodies for 1 h. The images were detected and analysed with Imaged Lab software (BioRad). GAPDH was selected to be the standard controls. HSP90 and Lamin B1 were served as reference proteins for cytosolic and nuclear fraction. All the antibody employed in this study are listed in Table S2.

### 2.7 Coimmunoprecipitation (CO‐IP)

Coimmunoprecipitation (CO‐IP) was performed using a Dynabeads™ Co‐Immunoprecipitation Kit (Thermo Scientific). The detailed procedures were operated according to the manufacturer's instructions. The protein complex was extracted by IP lysis buffer (5 × IP buffer, 100 mM NaCl, 2 mM MgCl_2_, and 1 mM DTT) with beads premixed with antibodies. The protein complex was separated from beads after several washes. The proteins were identified by immunoblots. All the antibody employed in this study are listed in Table S2.

### 2.8 RNA interference and Lentivirus infection

Small interfering RNAs (siRNA) were synthesized by GenePharma Company (Shanghai). Each gene was interfered with by three effective siRNAs to reduce off‐target effects. Transient transfection was performed by Lipofectamine 2000 Reagent (Invitrogen) in accordance with the standard protocol. The final efficiencies were detected by qRT‐PCR and western blotting. All siRNA sequences used in this study are listed in Table S3. The lentivirus (LV‐RanGAP1‐WT, LV‐RanGAP1‐K524R) were constructed by Bio link Company. HKF1 cells were chosen for stable RanGAP1 overexpression. 10^5^ cells were planted into a six‐well plate and infected at MOI of 50 for 24 h. Infections were supplemented with 3 mg/ml polybrene (Solarbio). The infection efficiency was evaluated by fluorescence microscopy, and HKFs were selected using 2 μg/ml puromycin (MCE) for 1 week.

### 2.9 Cell proliferation assay and wound healing assay

Cell proliferation ability was measured by Cell Counting Kit‐8 (CCK‐8, Dojindo Laboratories). HKFs were seeded into a 96‐well plate at 10^4^ cells per well. CCK‐8 solution was added to per well, and plate was incubated at 37°C for 2 h. The OD values were detected at 450 nm for 4 consecutive days with MRX II absorbance reader (Dynex Technologies). HKFs were planted in a little lattice (Servicebio) with 5 × 10^3^ per hole. After incubation overnight, the lattice was removed and created a shaped wound. The wound was washed with phosphate buffered saline (PBS) and photographed by a phase‐contrast microscope (Olympus) 24 and 48 h later.

### 2.10 Migration and invasion assays

Migration and invasion assays were performed with 8 μm pore size transwell chamber (Corning) with or without coating with Matrigel (BD Bioscience). HKFs were transfected with siRanGAP1 and siNC and seeded into the upper chamber with serum‐free medium. The bottom chamber was immersed in culture medium containing 12% FBS. After incubation for 48 h, the cells on the outside of membrane were fixed with methanol for 30 min and stained in a 0.5% crystal violet solution for 20 min. The cells were photographed and counted in five random fields under a microscope (Olympus) at a 100× magnification.

### 2.11 Dual‐luciferase reporter assay

Reporter plasmids SBE‐Luc and pGL‐hRluc (Promega) were used to measure TGF‐β‐induced transcription. HKFs were planted into a 96‐well plate at 5 × 10^4^ cells per well and co‐transfected with 100 ng of the SBE‐Luc and 50 nM siRanGAP1 and siNC. After 48 h transfection, cells were treated with TGF‐β1 (5 ng/ml) for 6 h. Then, cells were harvested to access the luciferase activity using Dual‐Glo Luciferase system (Promega). The fluorescein activity of the firefly was normalized to the expression to the Renilla luciferase in each sample. Each group was conducted in triplicate.

### 2.12 Statistical analysis

Statistical analysis was performed using SPSS 22.0 (SPSS Inc.) and GraphPad Prism 7.0 software (GraphPad Inc.). All experiments were repeated three times. Measure data were expressed as the mean ± SD. Two‐tail Student's *t*‐test was applied to compare quantitative data. A *p*‐value of 5% or lower is consider to be statistically significant. (*p < 0.05; **p < 0.01).

## 3 RESULTS

### 3.1 RanGAP1 acts as a functional partner of SUMOs in keloids

**FIGURE 1:**
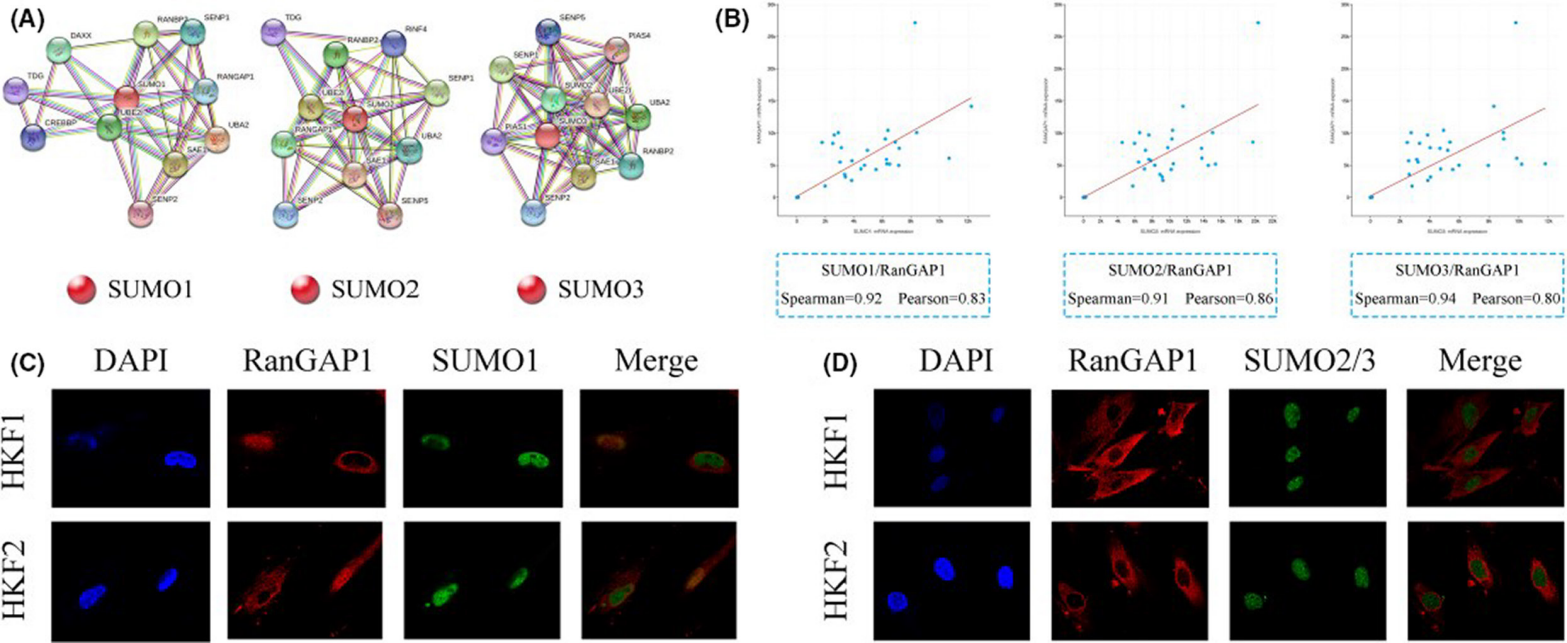
SUMOs and RanGAP1 are related in keloids. (A) The STRING database (http://www.stringdb.org/) predicted the top 10 functional partners of SUMO1, SUMO2 and SUMO3. (B) The figure shows the mRNA expression levels of SUMOs and RanGAP1 in 124 samples of skin‐related carcinoma with data available from cBioPortal (http://www.cbioportal.org/). The Spearman and Pearson correlation coefficients indicated that SUMOs and RanGAP1 were positively correlated in skin cancers.(C, D) Immunofluorescence double staining for SUMO1, SUMO2, SUMO3 and RanGAP1 was performed in HKF1 and HKF2 cells, and the results showed that SUMO1 and RanGAP1 were predominantly colocalized in the nucleus, especially in the cytoplasmic fibers of nuclear pore complexes. Images were taken with confocal microscopy (magnification 800×)

We predicted the proteins interacting with SUMOs with the STRING website. Hundreds of proteins were predicted to interact with SUMO1‐3 in Homo sapiens, the top of which are shown in Figure 1A. In addition to SUMO‐activating enzymes (SAE1 and UBA2), SUMO‐conjugating enzymes (UBE2I) and SENPs, which are involved in the processes of sumoylation and desumoylation, RanGAP1 was found to be closely related to SUMOs and had a high reliability score (Figure S1A). RanGAP1 was the first documented SUMO1 substrate and is one of the most prominent SUMO1 substrates to date.^20^ Moreover, RanGAP1 is highly expressed in skin, but its expression in keloids has not yet been reported (Figure S1B). Therefore, we selected RanGAP1 as a binding partner of SUMOs to explore the regulatory mechanism of sumoylation in keloids.

We analysed 124 samples of skin‐related carcinoma with data available from cBioPortal database and found significant positive correlations between the expression levels of RanGAP1 with SUMO1, SUMO2 and SUMO3 (Figure 1B). RanGAP1 and SUMOs expression was related in theses samples.

Normal skin and keloid tissues were stain with RanGAP1 and SUMOs. RanGAP1 was highly expressed in both samples, while the expression of SUMOs in keloid was higher than that in normal skin tissues (Figure S2). The immunofluorescence co‐staining results revealed that SUMOs and RanGAP1 had an obvious relationship in keloids. Confocal microscopy images further demonstrated that SUMO1 partially colocalized with RanGAP1 in human keloid fibroblasts (HKFs), predominantly at the cytoplasmic fibres of NPCs (Figure 1C,D). Altogether, our results indicate that RanGAP1 acts as a functional partner of SUMOs in keloids, and the positive regulation of sumoylation in keloids might be related to RanGAP1.

### 3.2 SUMO1 affects the nucleocytoplasmic distribution of Smad4 in the TGF‐β/Smad signalling pathway

**FIGURE 2:**
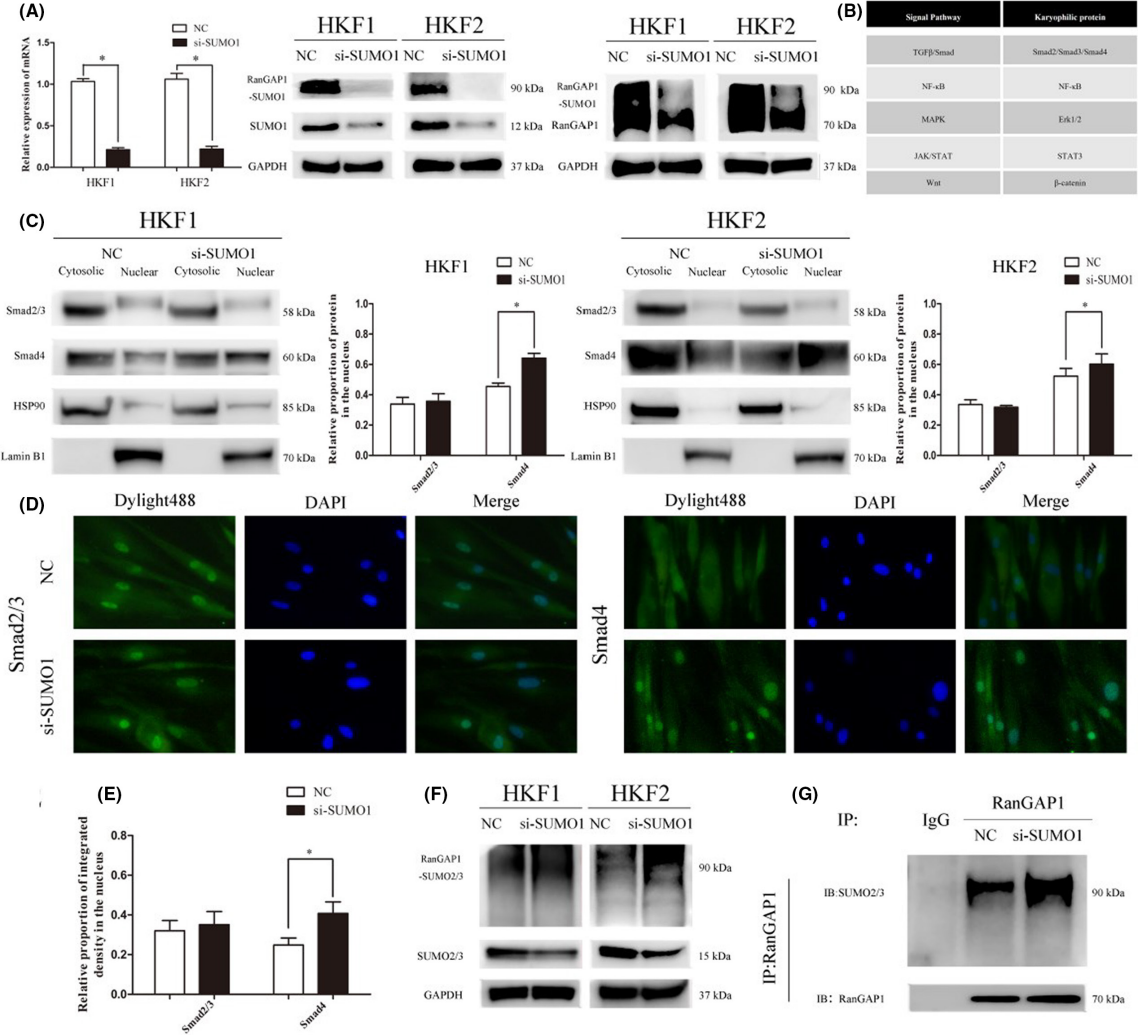
Inhibition of SUMO1 leads to the nuclear accumulation of Smad4 in HKFs. (A) qRT‐PCR and western blotting were performed to determine the silencing efficiency of siSUMO1 in HKF1 and HKF2 cells (left IB:SUMO1; right IB:RanGAP1). The expression level of RanGAP1*SUMO1 was also apparently decreased after silencing of SUMO1 (*p < 0.05). (B) The table shows the classical signaling pathways and their karyophilic proteins in keloids. (C) The nucleocytoplasmic expression levels of Smad2/3 and Smad4 in the TGF‐β/Smad signaling pathway were measured after extraction of nuclear and cytoplasmic proteins from HKF1 and HKF2 cells. HSP90 and Lamin B1 were reference proteins in the cytosol and nucleus. The histogram shows the relative proportion of protein distributed in the nucleus (*p < 0.05). (D, E) The intracellular localization of Smad2/3 and Smad4 protein was detected by immunofluorescence staining (magnification 600×). The histogram shows the relative proportion of fluorescence intensity in the nucleus (*p < 0.05). (F) The expression level of RanGAP1*SUMO2/3 was obviously upregulated in the siSUMO1 group, while the expression level of free SUMO2/3 was downregulated. (G) Coimmunoprecipitation showed an increased amount of RanGAP1 bound to SUMO2/3 in the siSUMO1 group compared with the negative control group

RanGAP1 is a GTPase‐activating protein for Ran, which is a key regulator of the Ran‐GTP/GDP cycle. Thus, we speculate that the regulation of sumoylation in keloids may be related to the role of RanGAP1 in nucleocytoplasmic transport of proteins in HKFs. We used small interfering RNA (siRNA) to knockdown the expression of SUMO1 in HKF1 and HKF2 cells. The silencing efficiency of siRNA was verified by qRT‐PCR and western blotting, and the content of RanGAP1*SUMO1 was also significantly decreased (Figure 2A). Next, we compared the nucleoplasmic distribution of karyophilic proteins, which enter the nucleus to perform functional roles, in several classical signalling pathways, such as TGF‐β/Smad, NF‐κB, MAPK, JAK/STAT and Wnt (Figure 2B). Most of the karyophilic proteins showed no significant difference in nuclear distribution when the expression of SUMO1 was decreased (Figure S3A). However, the proportion of Smad4 in the nucleus was significantly increased in HKF1 and HKF2 cells (Figure 2C). The immunofluorescence results further demonstrated that the proportion of Smad4 in the nucleus was significantly increased, while the distribution of Smad2/3 in the nucleus and cytoplasm did not change significantly (Figure 2D,E). We next upregulated SUMO1 levels in HKF1 cells by transfecting them with lentiviruses carrying a SUMO1 overexpression construct and found that the proportion of Smad4 distributed in the nucleus was decreased, which was consistent with the previous conclusion, although the overall content of Smad4 was increased (Figure S3B).

We also found that the proportion of RanGAP1 distributed at the cytoplasmic fibres of NPCs was significantly decreased when SUMO1 was silenced in HKFs. Previous studies have reported that SUMO1 mediates the targeting of RanGAP1 to the cytoplasmic fibres of NPCs.^24^ Although the results of western blotting (Figure 2F) and coimmunoprecipitation (Figure 2G) showed increased binding of SUMO2/3 and RanGAP1 after transfection with siSUMO1, the compensatory effect of SUMO2/3 on the nucleoplasmic distribution of Smad4 identified in the previous results remains to be further studied.^21^ Altogether, our results indicate that SUMO1 affects the nucleocytoplasmic distribution of Smad4, a member of the TGF‐β/Smad signalling pathway. A decrease in SUMO1 leads to the nuclear accumulation of Smad4.

### 3.3 SUMO1‐mediated nuclear accumulation of Smad4 is associated with RanGAP1

**FIGURE 3:**
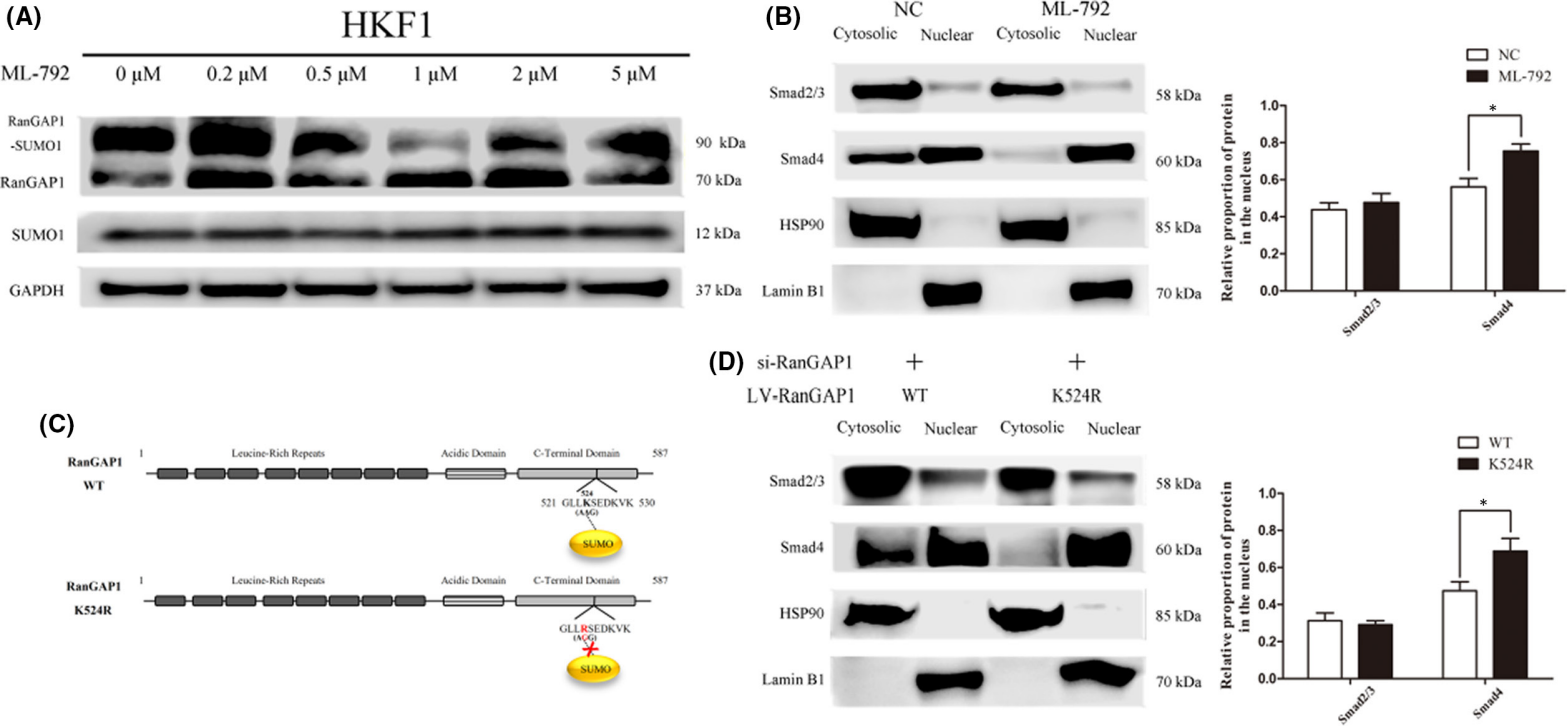
SUMO1 mediates the nuclear accumulation of Smad4 and is associated with RanGAP1. (A) HKF1 cells were treated with a certain concentration gradient of ML‐792 for 2 h, and the inhibition of SAEs by ML‐792 was assessed by western blotting for the RanGAP1‐SUMO1 complex and free SUMO1. (B) The nucleocytoplasmic expression levels of Smad2/3 and Smad4 were measured after treatment of HKF1 cells with 1 μM ML‐792 for 2 h. HSP90 and Lamin B1 were reference proteins in the cytosol and nucleus, respectively. The histogram shows the relative proportion of protein in the nucleus (*p < 0.05). (C) Structural motifs of the wild‐type and mutant RanGAP1 proteins. Both proteins contained eight leucine‐rich repeats and highly acidic stretches. Lysine 524 was replaced by arginine in mutant RanGAP1 to disrupt the sumoylation site of RanGAP1. (D) HKF1 cells with low endogenous RanGAP1 levels were transfected with wild‐type and mutant RanGAP1 constructs. The nucleocytoplasmic expression levels of Smad2/3 and Smad4 were measured. HSP90 and Lamin B1 were reference proteins in the cytosol and nucleus, respectively. The histogram shows the relative proportion of protein in the nucleus (*p < 0.05)

There are a number of studies on the effect of sumoylation on protein subcellular localization, but the specific effect of sumoylation on the nuclear localization of Smad4 remains to be studied. To further explore whether the nuclear localization of SUMO1 or sumoylation is related to RanGAP1, we used the SUMO inhibitor ML‐792, which is a potent, selective sumoylation inhibitor that specially targets SUMO activases. Previous studies have shown that ML‐792 has an inhibitory effect on the formation of RanGAP1*SUMO1.^25^ As shown in Figure 3A, we chose 1 μM as the concentration of ML‐792 to administer to HKFs (Figure 3A). The results showed that Smad4 accumulated in the nucleus of HKF1 cells after 2 h of treatment with 1 μM ML‐792, and the proportion of Smad4 in the nucleus increased significantly (Figure 3B).

Based on previous studies, lysine 524 is a sumoylation binding site in the C‐terminal region of RanGAP1. We designed a mutant by replacing lysine 524 with arginine to disrupt the sumoylation site of RanGAP1 (Figure 3C). After lentivirus construction, we transfected mutant RanGAP1 and wild‐type RanGAP1 into HKFs with low endogenous RanGAP1. The results showed that when RanGAP1 lacked the sumoylation site, the proportion of Smad4 in the nucleus increased significantly (Figure 3D). In summary, SUMO1‐mediated nuclear accumulation of Smad4 is associated with RanGAP1, and the level of RanGAP1*SUMO1 is directly related to the nucleocytoplasmic distribution of Smad4.

### 3.4 RanGAP1*SUMO1 affects the nuclear export of Smad4

**FIGURE 4:**
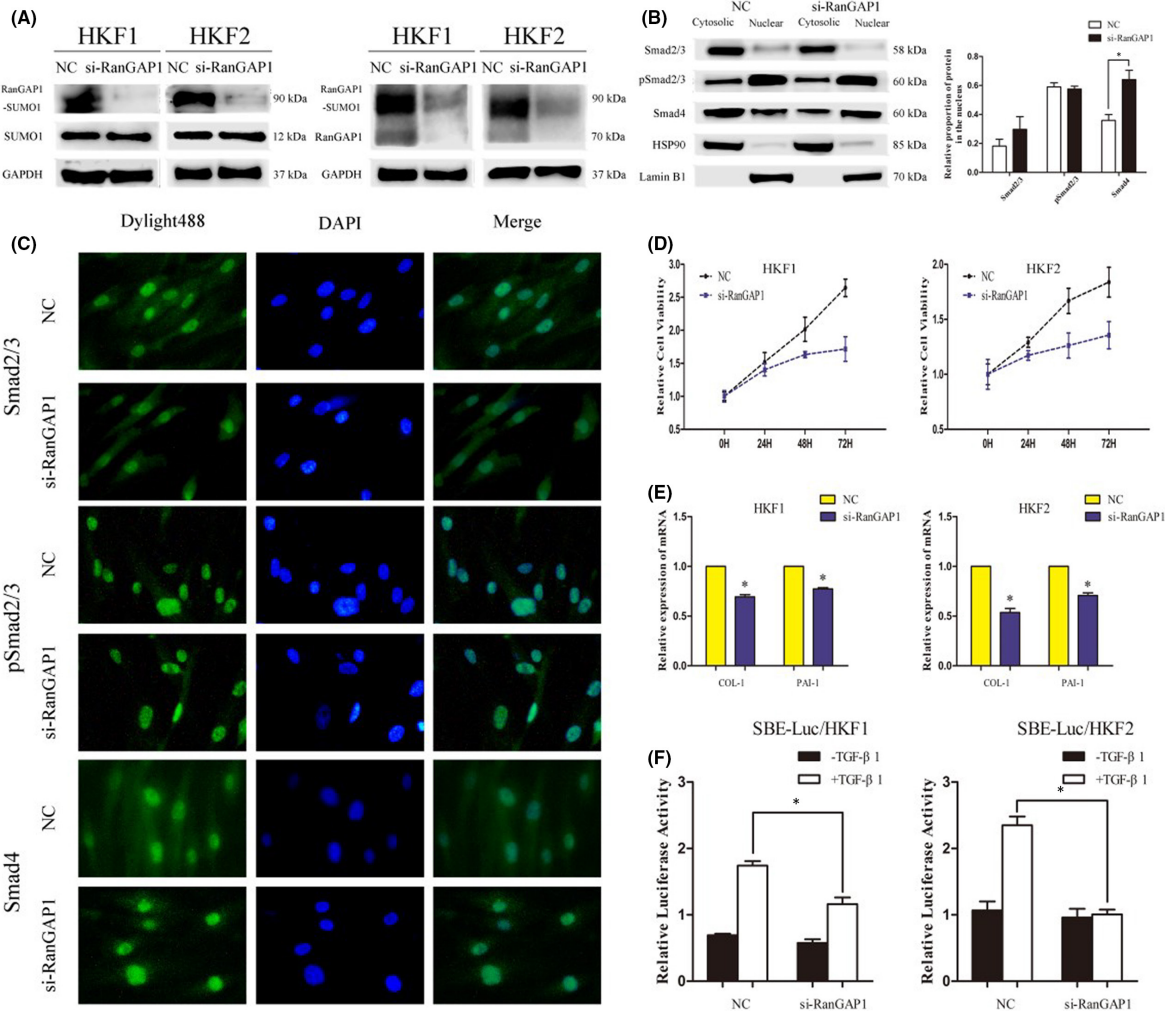
Inhibition of RanGAP1 leads to restriction of the nuclear export of Smad4 in HKFs. (A) Western blotting showed the silencing efficiency of siRanGAP1 in HKF1 and HKF2 cells (left IB:SUMO1; right IB:RanGAP1). The expression level of RanGAP1*SUMO1 was also apparently decreased after silencing of RanGAP1. (B) The nucleocytoplasmic expression levels of Smad2/3, pSmad2/3 and Smad4 were measured after transfection with siRanGAP1 in HKF1 cells. Both groups were treated with 5 ng/ml TGF‐β1 for 6 h. HSP90 and Lamin B1 were reference proteins in the cytosol and nucleus, respectively. (C) The intracellular localization of Smad2/3, pSmad2/3 and Smad4 protein was detected by immunofluorescence staining (magnification 600×). (D) The relative cell viability of the siRanGAP1 group was lower than that of the negative control group of HKF1 and HKF2 cells. The cell viability at 0 h was regarded as 1.0, and the cell viability was measured by CCK‐8 assay at 24, 48 and 72 h after transfection. (E) The relative mRNA expression levels of COL‐1 and PAI1 were measured by qRT‐PCR after transfection of HKF1 and HKF2 cells with siRanGAP1 (*p < 0.05). (F) SBE‐Luc reporter activity was measured in HKF1 and HKF2 cells transfected with siRanGAP1 and treated with 5 ng/ml TGF‐β1 for 6 h. Values and error bars represent the mean and standard deviation of three experiments, respectively (*p < 0.05)

To explore how RanGAP1*SUMO1 causes a change in the nuclear cytoplasmic distribution of Smad4, we used siRNA to knock down the expression of RanGAP1 in HKF1 and HKF2 cells. The silencing efficiency of the siRNA was verified by western blotting, and the content of RanGAP1*SUMO1 also was significantly decreased (Figure 4A). To further activate the TGF‐β/Smad signalling pathway and simulate the physiological condition of keloids, we treated HKFs with 5 ng/ml exogenous TGF‐β1 for 6 h for subsequent experiments (Figure S4). The results indicated that when the level of RanGAP1 was decreased, the proportion of Smad4 in the nucleus was significantly increased, while the proportions of Smad2/3 and p‐Smad2/3 in the nucleus did not change (Figure 4B). The immunofluorescence images also confirmed this result (Figure 4C, Figure S3C).

Our previous studies showed that sumoylation is positively regulated in keloids, but the regulatory role of RanGAP1*SUMO1 in keloids is still unknown. We used a CCK‐8 assay to investigate the effect of RanGAP1*SUMO1 on cell proliferation. The results revealed that downregulation of RanGAP1 suppressed the proliferation of HKF1 and HKF2 cells (Figure 4D), which might be related to the involvement of RanGAP1*SUMO1 in mitosis.^26^ Then, we investigated the role of RanGAP1 in the motility of HKFs. The results of the wound healing and transwell assays suggested that a decrease in RanGAP1 levels inhibited the motility of HKFs to a certain extent, but the difference was not significant (Figure S5). We selected COL and PAI as downstream genes of the Smad complex. The results showed that the mRNA and protein levels of COL‐1 and PAI‐1 were significantly decreased in siRanGAP1 HKFs (Figure 4E, Figure S3D). To further evaluate the role of RanGAP1*SUMO1 in the TGF‐β/Smad signalling pathway, we examined the effects of RanGAP1 on Smad complex‐mediated transcriptional activation using Smad‐dependent gene reporters. We performed experiments with a Smad‐binding element (SBE)‐Luc reporter, and silencing RanGAP1 in HKFs caused a decrease in TGF‐β1‐dependent transcription (Figure 4F), which suggested that the Smad4 accumulated in the nucleus did not participate in the Smad complex to regulate downstream genes and phenotypes. Altogether, our results suggest that the RanGAP1*SUMO1*‐mediated nuclear accumulation of Smad4 is due to its impact on nuclear export. Smad4 was unable to translocate into the cytoplasm, preventing continuous shuttling between the nucleus and the cytoplasm.

### 3.5 RanGAP1*SUMO1 functions as a disassembly machine for the export receptor CRM1 and Smad4

**FIGURE 5:**
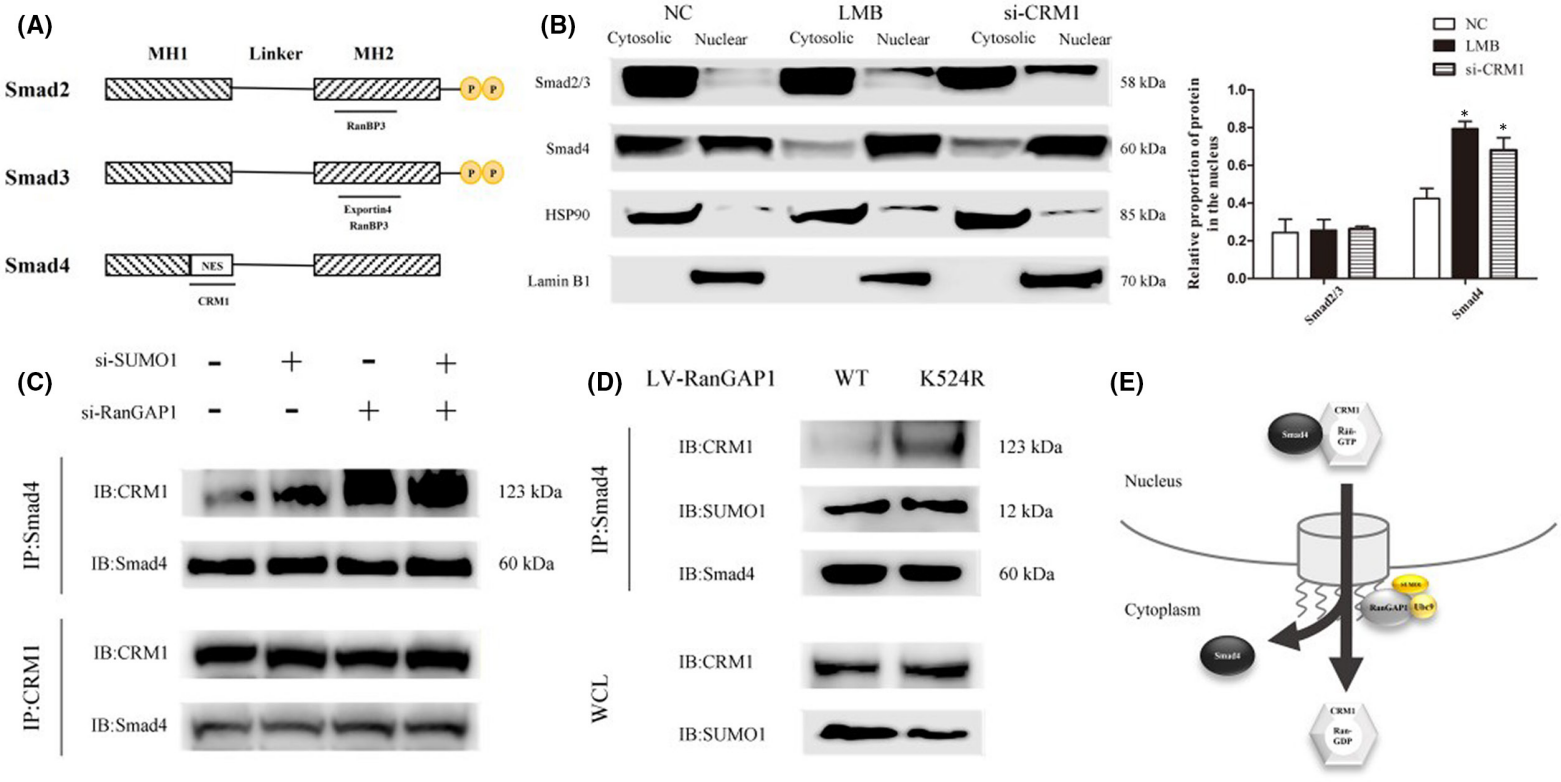
RanGAP1*SUMO1 mediates the dissociation of Smad4 and CRM1. (A) Structural motifs of the Smad2, Smad3 and Smad4 proteins. R‐Smad proteins and Smad4 share two conserved domains, the N‐terminal MH1 domain and the C‐terminal MH2 domain. These domains are connected by a proline‐rich linker region. The regions of Smad proteins that interact with exportin are shown. The position of the NES in Smad4 is also shown. (B) The nucleocytoplasmic expression levels of Smad2/3 and Smad4 were measured after transfection with siCRM1 and treatment with 50 nM leptomycin B for 12 h. The histogram shows the relative proportion of protein in the nucleus (*p < 0.05). (C) CRM1 was coimmunoprecipitated with Smad4 in HKFs. The binding of CRM1 and Smad4 was increased after transfection with siSUMO1 and siRanGAP1 (upper, second and third lane), which was consistent with the CRM1coimmunoprecipitation results. (D) Loss of the SUMO binding site in RanGAP1 made it more difficult to dissociate CRM1 and Smad4 (upper lane). The sumoylation of Smad4 was apparently unchanged (middle lane). (E) A schematic model illustrating our finding that RanGAP1*SUMO1 serves as a disassembly machine for the export receptor CRM1 and Smad4

Studies on Smad protein nuclear export have suggested that R‐Smad dephosphorylation leads to dissociation of Smad complexes in the nucleus and the export of monomeric Smad proteins to the cytoplasm. Smad2 and Smad3 require exportin 4 or RanBP3 for nuclear export, but the conserved sequence for nuclear export remains to be studied. However, the mechanism of Smad4 export from the nucleus has been shown to be mediated by CRM1,^27^ and this export relied on the canonical nuclear export signal (NES) in the N‐terminal part of the linker region of Smad4 (Figure 5A). Inhibition of CRM1 mediated nuclear export using CRM1 siRNA and inactivation of CRM1 by leptomycin B (LMB) resulted in nuclear accumulation of Smad4 in HKFs (Figure 5B). Coimmunoprecipitation showed increased binding of Smad4 and CRM1 after silencing of RanGAP1 and SUMO1. The results indicate that the effect of RanGAP1*SUMO1 on the nuclear export of Smad4 identified in the previous study was due to reduced dissociation of Smad4 and CRM1. RanGAP1 and SUMO1 played a synergistic role in this process, and RanGAP1 seemed to be more effective (Figure 5C). The loss of the SUMO binding site in RanGAP1 also limited the dissociation of Smad4 and CRM1, but it did not seem to influence the sumoylation of Smad4 in HKFs (Figure 5D). In summary, RanGAP1*SUMO1 mediates the dissociation of Smad4 and CRM1 and affects the nuclear export of Smad4. The role of sumoylation of Smad4 in nuclear export remains to be studied.

## 4 DISCUSSION

Sumoylation and desumoylation are dynamic, reversible processed that can be reversed by SENPs in vivo.^28^ For most substrates, SUMO modification is time‐dependent, which is necessary for the cycle of sumoylation, but it may cause bias for studies on the role of sumoylation.^29^ For example, hypoxia‐inducible factor 1α (HIF‐1α) is modified by SUMO1 and can still be hydrolysed by SENP1 under hypoxic conditions. Two independent studies reported that sumoylation can aid the stabilization of the HIF‐1α protein structure by adding SUMO1 to lysines 391 and 477 in the HIF‐1α oxygen‐dependent degradation region to antagonize ubiquitination.^30^, ^31^ However, Cheng et al.^18^ refuted this conclusion by suggesting that the stability of HIF‐1α decreased when the level of sumoylation was enhanced by knocking down SENP1. In our previous study, when we inhibited sumoylation and desumoylation in keloids by silencing SUMO1 and SENP1, the regulatory effect on HKFs was related to the content of SUMO complexes but not free SUMO.^17^ Therefore, we hypothesized that SUMO proteins can exist in complexes. When hydrolase is absent, although the proportion of free SUMO decreases, the proportion of SUMO complexes increases. This also explains why the effect of hydrolase knockdown and the effect of SUMO knockdown are contradictory in sumoylation studies. A SUMO‐complex band was easily observed near 90 kDa, which was further confirmed to be the RanGAP1*SUMO1 form. The functional partners of SUMO proteins predicted by STRING also suggest that there is a close relationship between SUMO proteins and RanGAP1. In addition, RanGAP1 is covalently modified by SUMO1 and targeted to NPCs, which remain stably associated with the nuclear envelope.^26^ We chose to investigate RanGAP1, as a chaperone of SUMO proteins, in our study of the effect of sumoylation on keloids.

RanGAP1 was initially purified as a homodimer of 70 kDa subunits located in the cytoplasm that specifically enhanced the rate of Ran‐GTP hydrolysis by three orders of magnitude.^1^ Ran is a nuclear Ras‐like GTPase that is converted to its GTP‐bound form in the nucleus and hydrolysed to its GDP‐bound form in the cytoplasm.^32^ The concentration gradient of GTP/GDP is an important factor regulating nucleocytoplasmic transport. Later, RanGAP1 was found to be highly concentrated at NPCs and to form a stable complex with RanBP2 with SUMO1 modification.^20^, ^33^ Our confocal microscopy images also showed that RanGAP1 was mainly distributed in the cytoplasm and enriched in the nuclear envelope in HKFs. Therefore, RanGAP1 is present in two forms: one that is cytoplasmic and another that is concentrated at the cytoplasmic fibres of NPCs. The western blotting results showed 70 kDa unmodified RanGAP1 and 90 kDa modified RanGAP1, as well as the 10 kDa free SUMO1 and 90 kDa modified SUMO1. RanGAP1*SUMO1 plays essential roles in nucleocytoplasmic transport, mitotic spindle formation, checkpoint control and postmitotic nuclear envelope reassembly.^34^, ^35^, ^36^ The NPC‐associated form of RanGAP1*SUMO1 is targeted to RanBP2 and interacts with RanBP2 and UBC9. The RanBP2/RanGAP1*SUMO1/UBC9 complex is a multisubunit E3 ligase rather than an E2‐E3 complex. This difference is due to the formation of a stable UBC9‐containing E3 ligase complex and a lack of the typical E2‐E3 interaction^22^ The complex can not only recruit proteins as substrates for sumoylation but also fine tune the transport machinery.^37^ We downregulated the expression of RanGAP1*SUMO1 by silencing SUMO1 and RanGAP1 and compared the nucleoplasmic distribution of karyophilic proteins in several classical signalling pathways via nucleocytoplasmic protein extraction and immunofluorescence staining. The results indicated that RanGAP1*SUMO1 affects the nucleocytoplasmic distribution of Smad4, a member of the TGF‐β/Smad signalling pathway.

Mediation of nucleocytoplasmic transport by sumoylation has been extensively documented: protein sumoylation effects on the physical properties of cargo proteins and/or regulates the functions of components of the transport machinery.^38^ The effects of sumoylation on the nucleocytoplasmic transport of cargo proteins include four aspects: export inhibition, export stimulation, import inhibition and import stimulation.^39^, ^40^, ^41^ For example, SUMO proteins induce colocalization of ATF7, which interacts with NPC components, leading to increased residence time of ATF7 at nuclear envelope and ultimately a slower rate of import.^42^ Sumoylation of p53 dramatically increases its nuclear export by facilitating disassembly of the transport complex and cargo release into the cytoplasm.^43^ Smad4 has been reported to be endogenously modified by SUMO1 in Hela cells and human ovarian cells, and lysine 159 is the main sumoylation site.^44^ Furthermore, Smad4 was also found to strongly interact with UBC9 in yeast.^45^ Shimada et al.^46^ proposed that UBC9 affects the nuclear accumulation of Smad1 and Smad4 in the BMP signalling pathway. In the present study, we showed that silencing SUMO1 and RanGAP1 resulted in nuclear accumulation of Smad4, a member of the TGF‐β/Smad signalling pathway, while Smad2/3 was not affected. Therefore, we suggest that the RanBP2/RanGAP1*SUMO1/UBC9 complex located at NPCs is the key factor that mediates the effect of sumoylation on the nucleocytoplasmic distribution of Smad4. In other words, sumoylation affects the nucleocytoplasmic transport of Smad4 because it regulates the functions of components of the transport machinery. Our results indicate that the sumoylation of Smad4 is not significantly affected by the nuclear accumulation of Smad4. The effect of Smad4 sumoylation on the nucleocytoplasmic transport of Smad4 as a cargo protein remains to be further investigated.

Generally, the positive regulatory function of Smad4 should be enhanced by its accumulation in the nucleus and the increased Smad4 content in the nucleus. However, when RanGAP1 was knocked down, the nuclear accumulation of Smad4 induced negative regulation in HKFs, and the same results were observed when SUMO1 was knocked down. Therefore, we propose that RanGAP1*SUMO1‐mediated nuclear accumulation of Smad4 affects its nuclear export such that Smad4 cannot translocate into the cytoplasm to continuously shuttle between the nucleus and the cytoplasm, which leads to negative regulation in HKFs after knockdown of RanGAP1*SUMO1. It is clear that the nucleocytoplasmic distribution of Smads is not static, but instead the Smad proteins are continuously shuttled between the nucleus and the cytoplasm in the absence and presence of the TGF‐β signal.^27^ The bulk Smad proteins are not degraded in the nucleus in response to a signal but rather engage in highly dynamic shuttling between the nucleus and the cytoplasm.^47^ Smad4 shuttling involves R‐Smad dephosphorylation, which leads to dissociation of Smad complexes in the nucleus and export of monomeric Smad proteins to the cytoplasm.^48^ The mechanism of Smad4 export from the nucleus was identified to be mediated by CRM1. The critical nuclear export signal in the N‐terminal part of Smad4 is necessary for the nuclear export of Smad4. Our experiments with CRM1 siRNA and LMB indicate that nuclear export of Smad4 is CRM1‐dependent. RanGAP1 mediated the disassembly of both trimeric export complexes and recycling of the import/Ran‐GTP complex.^49^ Moreover, as most SUMO proteins are found within the nucleus, sumoylation is strategically positioned to regulate nuclear export.^38^ It has been reported that the RanBP2/RanGAP1*SUMO1/UBC9 complex functions as an autonomous disassembly machine with a preference for the export receptor CRM1.^50^ In the present study, when the content of RanGAP1*SUMO1 was decreased, the binding of Smad4 and CRM1 significantly increased, which indicates RanGAP1*SUMO1 mediates the dissociation of Smad4 and CRM1 in nuclear export, as shown in Figure 5E.

## CONFLICT OF INTEREST

The authors declare that they have no conflicts of interest with the contents of this article.

## AUTHOR CONTRIBUTION

**Xiaohu Lin:** Conceptualization (equal); Data curation (equal); Formal analysis (equal); Writing – original draft (equal); Writing – review & editing (equal). **Qianqian Pang:** Formal analysis (equal). **Jie Hu:** Conceptualization (equal); Data curation (equal). **Jiaqi Sun:** Data curation (equal); Writing – original draft (equal). **Siya Dai:** Resources (equal). **Yijia Yu:** Funding acquisition (equal); Resources (equal). **Jinghong Xu:** Funding acquisition (equal); Writing – review & editing (equal).

## Supporting information

Supporting Information

## Data Availability

The data that support the findings of this study are openly available.

## References

[1] Matunis M , Coutavas E , Blobel G . A novel ubiquitin‐like modification modulates the partitioning of the Ran‐GTPase‐activating protein RanGAP1 between the cytosol and the nuclear pore complex. J Cell Biol. 1996;135:1457‐1470. 10.1083/jcb.135.6.1457 8978815 PMC2133973

[2] Tufegdžić Vidaković A , Mitter R , Kelly G , et al. Regulation of the RNAPII pool is integral to the DNA damage response. Cell. 2020;180(6):1245‐1261.e21. 10.1016/j.cell.2020.02.009 32142654 PMC7103762

[3] Hendriks I , Vertegaal A . A comprehensive compilation of SUMO proteomics. Nat Rev Mol Cell Biol. 2016;17(9):581‐595. 10.1038/nrm.2016.81 27435506

[4] Seeler J , Dejean A . Nuclear and unclear functions of SUMO. Nat Rev Mol Cell Biol. 2003;4(9):690‐699. 10.1038/nrm1200 14506472

[5] Zhao J . Sumoylation regulates diverse biological processes. Cell Mol Life Sci. 2007;64(23):3017‐3033. 10.1007/s00018-007-7137-4 17763827 PMC7079795

[6] Yang S , Sharrocks A . SUMO promotes HDAC‐mediated transcriptional repression. Mol Cell. 2004;13(4):611‐617. 10.1016/s1097-2765(04)00060-7 14992729

[7] Seeler J , Dejean A . SUMO and the robustness of cancer. Nat Rev Cancer. 2017;17(3):184‐197. 10.1038/nrc.2016.143 28134258

[8] Bartek J , Hodny Z . SUMO boosts the DNA damage response barrier against cancer. Cancer Cell. 2010;17(1):9‐11. 10.1016/j.ccr.2009.12.030 20129245

[9] Bergink S , Jentsch S . Principles of ubiquitin and SUMO modifications in DNA repair. Nature. 2009;458(7237):461‐467. 10.1038/nature07963 19325626

[10] Geiss‐Friedlander R , Melchior F . Concepts in sumoylation: a decade on. Nat Rev Mol Cell Biol. 2007;8(12):947‐956. 10.1038/nrm2293 18000527

[11] Liang Y , Lee C , Yao Y , Lai C , Schmitz M , Yang W . SUMO5, a novel Poly‐SUMO isoform, regulates PML nuclear bodies. Sci Rep. 2016;6:26509. 10.1038/srep26509 27211601 PMC4876461

[12] Hickey C , Wilson N , Hochstrasser M . Function and regulation of SUMO proteases. Nat Rev Mol Cell Biol. 2012;13(12):755‐766. 10.1038/nrm3478 23175280 PMC3668692

[13] Mustfa S , Singh M , Suhail A , et al. SUMOylation pathway alteration coupled with downregulation of SUMO E2 enzyme at mucosal epithelium modulates inflammation in inflammatory bowel disease. Open Biol. 2017;7(6):170024. 10.1098/rsob.170024 28659381 PMC5493774

[14] Tomasi M , Ryoo M , Ramani K , et al. Methionine adenosyltransferase α2 sumoylation positively regulate Bcl‐2 expression in human colon and liver cancer cells. Oncotarget. 2015;6(35):37706‐37723. 10.18632/oncotarget.5342 26416353 PMC4741959

[15] Liu Y , Zhao D , Qiu F , et al. Manipulating PML SUMOylation via silencing UBC9 and RNF4 regulates cardiac fibrosis. Mol Ther. 2017;25(3):666‐678. 10.1016/j.ymthe.2016.12.021 28143738 PMC5363217

[16] Ikeda M , Naitoh M , Kubota H , et al. Elastic fiber assembly is disrupted by excessive accumulation of chondroitin sulfate in the human dermal fibrotic disease, keloid. Biochem Biophys Res Comm. 2009;390(4):1221‐1228. 10.1016/j.bbrc.2009.10.125 19879246

[17] Lin X , Wang Y , Jiang Y , et al. Sumoylation enhances the activity of the TGF‐β/SMAD and HIF‐1 signaling pathways in keloids. Life Sci. 2020;255:117859. 10.1016/j.lfs.2020.117859 32474020

[18] Cheng J , Kang X , Zhang S , Yeh E . SUMO‐specific protease 1 is essential for stabilization of HIF1alpha during hypoxia. Cell. 2007;131(3):584‐595. 10.1016/j.cell.2007.08.045 17981124 PMC2128732

[19] Bischoff F , Klebe C , Kretschmer J , Wittinghofer A , Ponstingl H . RanGAP1 induces GTPase activity of nuclear Ras‐related Ran. Proc Natl Acad Sci USA. 1994;91(7):2587‐2591. 10.1073/pnas.91.7.2587 8146159 PMC43414

[20] Mahajan R , Delphin C , Guan T , Gerace L , Melchior F . A small ubiquitin‐related polypeptide involved in targeting RanGAP1 to nuclear pore complex protein RanBP2. Cell. 1997;88(1):97‐107. 10.1016/s0092-8674(00)81862-0 9019411

[21] Evdokimov E , Sharma P , Lockett S , Lualdi M , Kuehn M . Loss of SUMO1 in mice affects RanGAP1 localization and formation of PML nuclear bodies, but is not lethal as it can be compensated by SUMO2 or SUMO3. J Cell Sci. 2008;121:4106‐4113. 10.1242/jcs.038570 19033381

[22] Werner A , Flotho A , Melchior F . The RanBP2/RanGAP1*SUMO1/Ubc9 complex is a multisubunit SUMO E3 ligase. Mol Cell. 2012;46(3):287‐298. 10.1016/j.molcel.2012.02.017 22464730

[23] Sakin V , Richter S , Hsiao H , Urlaub H , Melchior F . Sumoylation of the GTPase ran by the RanBP2 SUMO E3 ligase complex. J Biol Chem. 2015;290(39):23589‐23602. 10.1074/jbc.M115.660118 26251516 PMC4583052

[24] Matunis M , Wu J , Blobel G . SUMO‐1 modification and its role in targeting the Ran GTPase‐activating protein, RanGAP1, to the nuclear pore complex. J Cell Biol. 1998;140(3):499‐509. 10.1083/jcb.140.3.499 9456312 PMC2140169

[25] He X , Riceberg J , Soucy T , et al. Probing the roles of SUMOylation in cancer cell biology by using a selective SAE inhibitor. Nat Chem Biol. 2017;13(11):1164‐1171. 10.1038/nchembio.2463 28892090

[26] Swaminathan S , Kiendl F , Körner R , Lupetti R , Hengst L , Melchior F . RanGAP1*SUMO1 is phosphorylated at the onset of mitosis and remains associated with RanBP2 upon NPC disassembly. J Cell Biol. 2004;164(7):965‐971. 10.1083/jcb.200309126 15037602 PMC2172064

[27] Hill C . Nucleocytoplasmic shuttling of Smad proteins. Cell Res. 2009;19(1):36‐46. 10.1038/cr.2008.325 19114992

[28] Drag M , Mikolajczyk J , Krishnakumar I , Huang Z , Salvesen G . Activity profiling of human deSUMOylating enzymes (SENPs) with synthetic substrates suggests an unexpected specificity of two newly characterized members of the family. Biochem J. 2008;409(2):461‐469. 10.1042/bj20070940 17916063

[29] Kroonen J , Vertegaal A . Targeting SUMO signaling to wrestle cancer. Trends Cancer. 2020;7(6):496‐510. 10.1016/j.trecan.2020.11.009 33353838

[30] Carbia‐Nagashima A , Gerez J , Perez‐Castro C , et al. RSUME, a small RWD‐containing protein, enhances SUMO conjugation and stabilizes HIF‐1alpha during hypoxia. Cell. 2007;131(2):309‐323. 10.1016/j.cell.2007.07.044 17956732

[31] Bae S , Jeong J , Park J , et al. Sumoylation increases HIF‐1alpha stability and its transcriptional activity. Biochem Biophys Res Comm. 2004;324(1):394‐400. 10.1016/j.bbrc.2004.09.068 15465032

[32] Pemberton L , Paschal B . Mechanisms of receptor‐mediated nuclear import and nuclear export. Traffic. 2005;6(3):187‐198. 10.1111/j.1600-0854.2005.00270.x 15702987

[33] Pichler A , Gast A , Seeler J , Dejean A , Melchior F . The nucleoporin RanBP2 has SUMO1 E3 ligase activity. Cell. 2002;108(1):109‐120. 10.1016/s0092-8674(01)00633-x 11792325

[34] Okada N , Sato M . Spatiotemporal regulation of nuclear transport machinery and microtubule organization. Cells. 2015;4(3):406‐426. 10.3390/cells4030406 26308057 PMC4588043

[35] Roscioli E , Di Francesco L , Bolognesi A , et al. Importin‐β negatively regulates multiple aspects of mitosis including RANGAP1 recruitment to kinetochores. J Cell Biol. 2012;196(4):435‐450. 10.1083/jcb.201109104 22331847 PMC3283988

[36] Clarke P , Zhang C . Spatial and temporal coordination of mitosis by Ran GTPase. Nat Rev Mol Cell Biol. 2008;9(6):464‐477. 10.1038/nrm2410 18478030

[37] von Appen A , Kosinski J , Sparks L , et al. In situ structural analysis of the human nuclear pore complex. Nature. 2015;526(7571):140‐143. 10.1038/nature15381 26416747 PMC4886846

[38] Ptak C , Wozniak R . SUMO and nucleocytoplasmic transport. Adv Exp Med Biol. 2017;963:111‐126. 10.1007/978-3-319-50044-7_7 28197909

[39] Du J , Bialkowska A , McConnell B , Yang V . SUMOylation regulates nuclear localization of Krüppel‐like factor 5. J Biol Chem. 2008;283(46):31991‐32002. 10.1074/jbc.M803612200 18782761 PMC2581587

[40] Rosas‐Acosta G , Wilson V . Identification of a nuclear export signal sequence for bovine papillomavirus E1 protein. Virology. 2008;373(1):149‐162. 10.1016/j.virol.2007.12.017 18201744 PMC2292128

[41] Vethantham V , Rao N , Manley J . Sumoylation regulates multiple aspects of mammalian poly(A) polymerase function. Genes Dev. 2008;22(4):499‐511. 10.1101/gad.1628208 18281463 PMC2238671

[42] Hamard P , Boyer‐Guittaut M , Camuzeaux B , et al. Sumoylation delays the ATF7 transcription factor subcellular localization and inhibits its transcriptional activity. Nucleic Acids Res. 2007;35(4):1134‐1144. 10.1093/nar/gkl1168 17264123 PMC1851647

[43] Santiago A , Li D , Zhao L , Godsey A , Liao D . p53 SUMOylation promotes its nuclear export by facilitating its release from the nuclear export receptor CRM1. Mol Biol Cell. 2013;24(17):2739‐2752. 10.1091/mbc.E12-10-0771 23825024 PMC3756925

[44] Lin X , Liang M , Liang Y , Brunicardi F , Melchior F , Feng X . Activation of transforming growth factor‐beta signaling by SUMO‐1 modification of tumor suppressor Smad4/DPC4. J Biol Chem. 2003;278(21):18714‐18719. 10.1074/jbc.M302243200 12621041

[45] Lin X , Liang M , Liang Y , Brunicardi F , Feng X . SUMO‐1/Ubc9 promotes nuclear accumulation and metabolic stability of tumor suppressor Smad4. J Biol Chem. 2003;278(33):31043‐31048. 10.1074/jbc.C300112200 12813045

[46] Shimada K , Suzuki N , Ono Y , Tanaka K , Maeno M , Ito K . Ubc9 promotes the stability of Smad4 and the nuclear accumulation of Smad1 in osteoblast‐like Saos‐2 cells. Bone. 2008;42(5):886‐893. 10.1016/j.bone.2008.01.009 18321803

[47] Seoane J , Gomis R . TGF‐β family signaling in tumor suppression and cancer progression. Cold Spring Harb Perspect Biol. 2017;9(12):a022277. 10.1101/cshperspect.a022277 28246180 PMC5710110

[48] Schmierer B , Hill C . TGFbeta‐SMAD signal transduction: molecular specificity and functional flexibility. Nat Rev Mol Cell Biol. 2007;8(12):970‐982. 10.1038/nrm2297 18000526

[49] Forbes D , Travesa A , Nord M , Bernis C . Nuclear transport factors: global regulation of mitosis. Curr Opin Cell Biol. 2015;35:78‐90. 10.1016/j.ceb.2015.04.012 25982429 PMC4529785

[50] Ritterhoff T , Das H , Hofhaus G , Schröder R , Flotho A , Melchior F . The RanBP2/RanGAP1*SUMO1/Ubc9 SUMO E3 ligase is a disassembly machine for Crm1‐dependent nuclear export complexes. Nat Commun. 2016;7:11482. 10.1038/ncomms11482 27160050 PMC4866044

