# Supplementary material for: SUMOylation mediates the disassembly of the Smad4 nuclear export complex via RanGAP1 in KELOIDS
Source: J Cell Mol Med. 2023 Mar 14;27(8):1045–55. doi: 10.1111/jcmm.17216 (PMC10098277; doi:10.1111/jcmm.17216)
Supplement: Supplementary file 1 — Supporting Information [file JCMM-27-1045-s001.pdf]

SUPPLEMENTAL INFORMATION

A

Predicted Functional Partners						
	SUMO1		SUMO2		SUMO3	
	proteins	score	proteins	score	proteins	score
1	UBA2	0.999	SAE1	0.999	UBE2I	0.998
2	SAE1	0.999	UBE2I	0.999	SAE1	0.997
3	RANBP2	0.999	SENP1	0.999	SENP1	0.995
4	UBE2I	0.999	RANBP2	0.998	UBA2	0.991
5	DAXX	0.999	RANGAP1	0.997	SUMO2	0.987
6	SENP1	0.999	UBA2	0.995	RANBP2	0.986
7	RANGAP1	0.998	SENP2	0.995	SENP2	0.980
8	CREBBP	0.997	RNF4	0.994	SENP5	0.980
9	TDG	0.996	TDG	0.993	PIAS1	0.979
10	SENPQ	0.995	SENP5	0.991	PIAS4	0.974

B

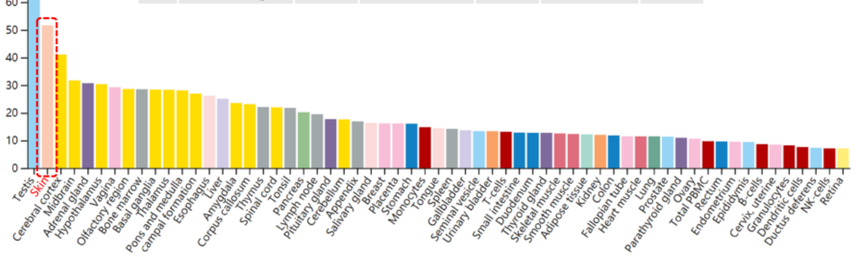

Fig. S1. (A) The table shows the reliability score of each protein predicted to be SUMO functional partner. The scores were based on the association between partner proteins and SUMO proteins, which was determined based on coexpression, gene fusion, cooccurence data, databases and experiments. (B) Data from The Human Protein Atlas (http://www.proteinatlas.org/) were used to determine the relative expression of RanGAP1 in various organs. RanGAP1 is highly expressed in skin.

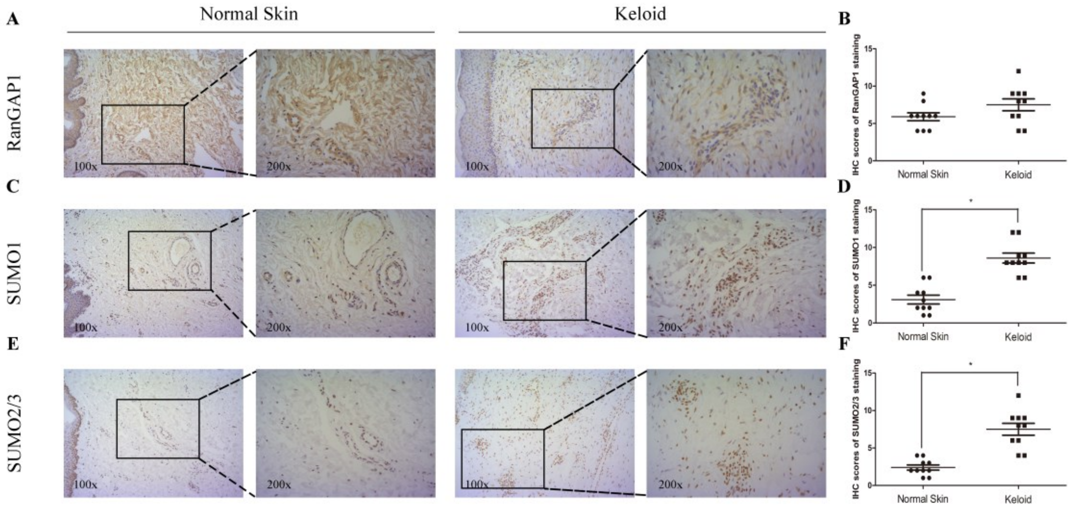

Fig. S2. (A, C, E) The expression levels of RanGAP1 and SUMOs. SUMO1 and SUMO2/3 are significantly higher in keloid tissues than they are in normal skin tissues (magnification x100 and x200). (B, D, F) IHC scores of 10 pairs of normal and keloid tissues (*P<0.05).

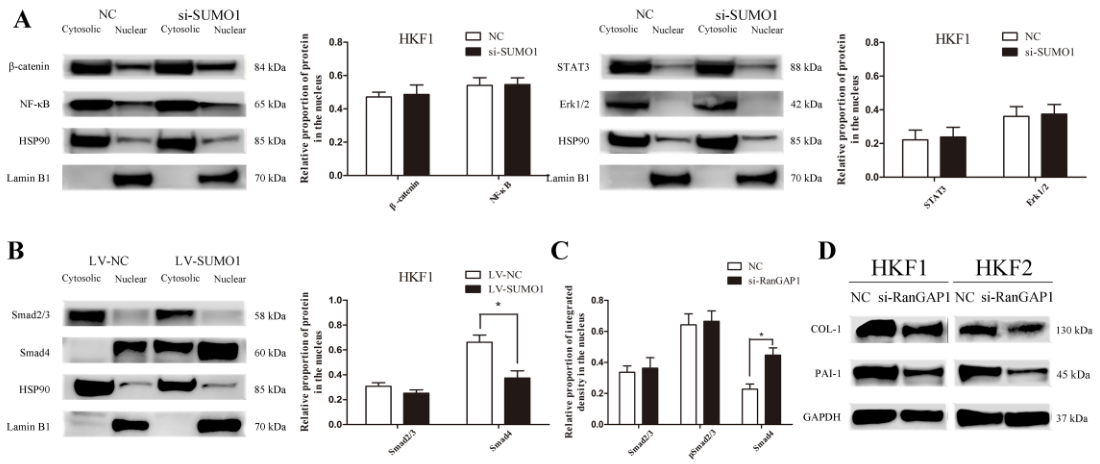

Fig. S3. (A) The nucleocytoplasmic expression levels of NF-κB, Erk1/2, STAT3 and β-catenin were measured after extraction of nuclear and cytoplasmic protein from in HKF1 cells. The histogram shows the relative proportion of protein in the nucleus. (B) The nucleocytoplasmic expression levels of Smad2/3 and Smad4 were measured in the LV-NC and LV-SUMO1 groups, and the proportion of Smad4 in the nucleus was significantly decreased (*P<0.05). (C) The histogram shows the relative proportion of fluorescence intensity in the nucleus (*P<0.05). (D) The protein levels of COL-1 and PAI1 were measured after transfection of HKF1 and HKF2 cells with siRanGAP1.

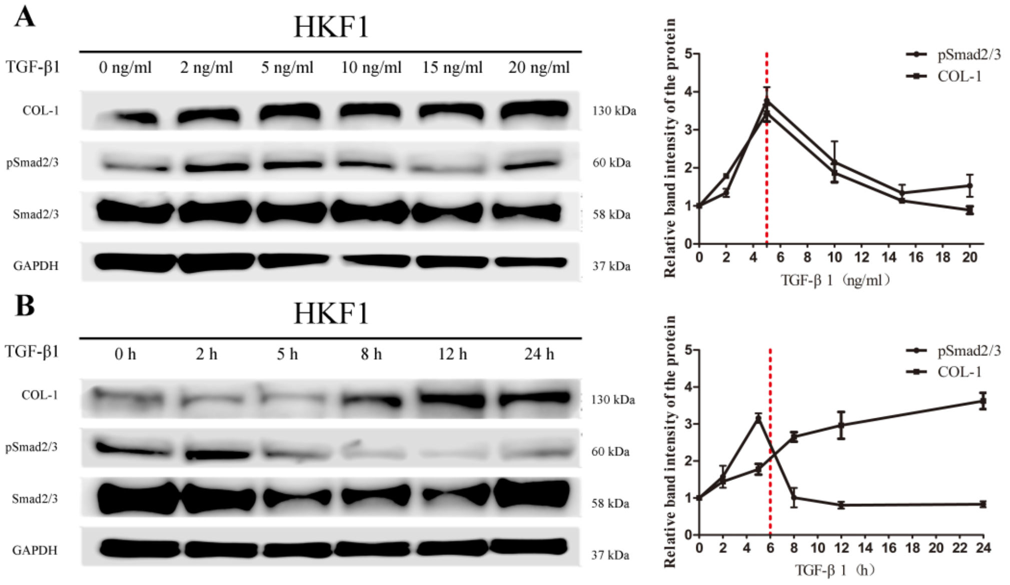

Fig. S4. (A) HKF1 cells were treated a specific concentration gradient of TGF-β1 for 4 h, and the activation of the TGF-β/Smad signaling pathway was assessed by western blotting for pSmad2/3, Smad2/3 and COL-1. The effect of different TGF-β1 concentrations was evaluated by assessing the phosphorylation of Smad2/3 and the relative expression of COL-1. (B) HKF1 cells were treated with 5 ng/ml TGF-β1 for a specific time period, and activation of the TGF-β/Smad signaling pathway was assessed by western blotting for pSmad2/3, Smad2/3 and COL-1. The effect of TGF-β1 exposure time was evaluated by assessing the phosphorylation of Smad2/3 and the relative expression of COL-1.

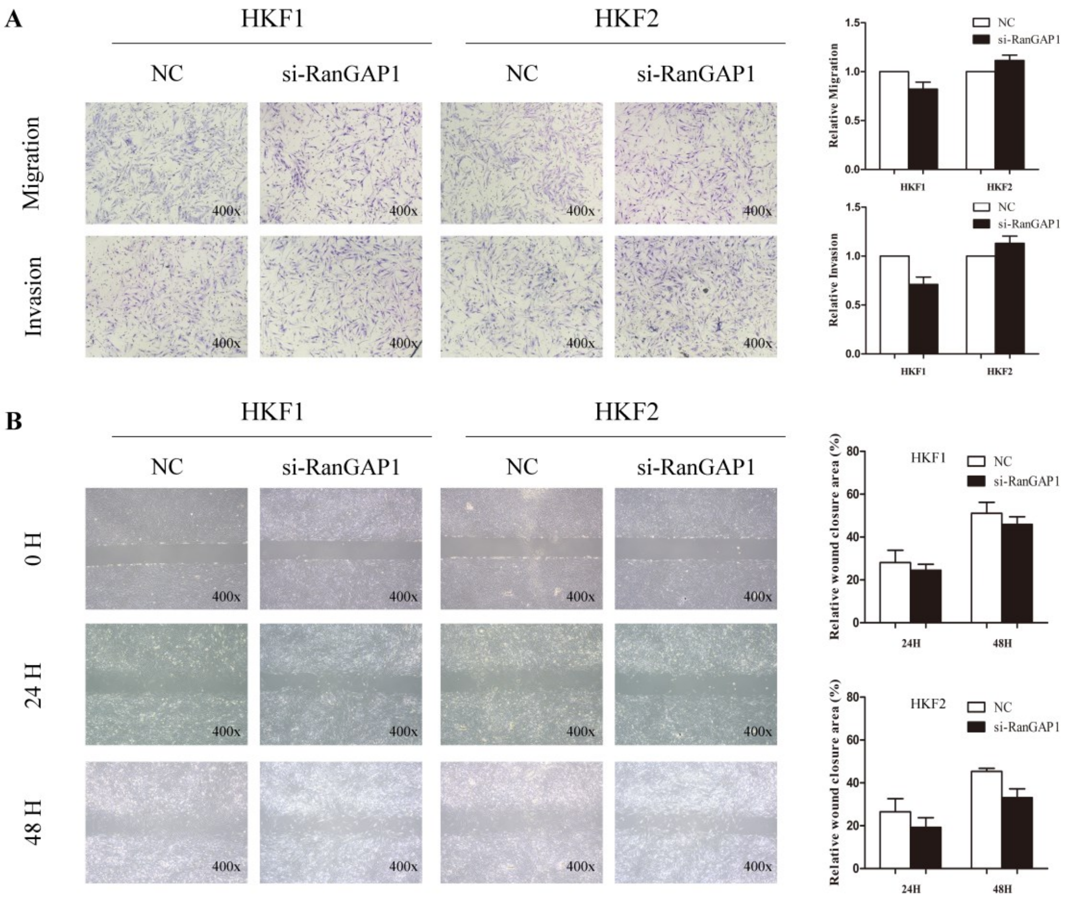

Fig. S5. (A) HKF1 and HKF2 cells were transfected with siRanGAP1 or NC for 24 h. Transwell assays were used to evaluate changes in cell migration and invasion (magnification 400x). (B) Wound healing assays were used to evaluate the change in cell motility. Representative images are shown, and there were no significant differences between the NC group and the siRanGAP1 group (magnification 400x).

Supplementary Table-1

The information of patients and specimens

Information		Keloid
Number		40
Sex	Male	15 (37.5%)
	Female	25 (62.5%)
Age(yrs)		24.63
Side of Biopsy		Earlobe: 20(50.0%)
		Auricle: 10(25.0%)
		Back: 4(10.0%)
		Shoulder: 3(7.5%)
		Chest: 2 (5.0%)
		Forehead: 1(2.5%)

Supplementary Table-2

Antibody used in this study

Gene	Brand	Cat No	Gene	Brand	Cat No
SUMO1	Abcam	ab32058	PAI-1	Abcam	ab222754
SUMO2+SUMO3	Abcam	ab3742	COL I	Abcam	ab34710
RanGAP1	Abcam	ab92360	CRM1	Abcam	ab191081
Smad2+Smad3	Abcam	ab202445	β -catenin	Proteintech	51067-2-AP
pSmad2+pSmad3	CST	#8828	NF-κB	Abcam	ab16502
Smad4	Abcam	ab230815	STAT3	CST	#12640
HSP90	Abcam	ab53497	Erk1/2	Abcam	ab17942
Lamin B1	Proteintech	12987-1-AP	GAPDH	Bioss	bs10900R

Supplementary Table-3

Oligonucleotide primers used in qRT-pcr analysis

Oligo Name	Sequence(5' to3')	Oligo Name	Sequence(5' to3')
SUMO1-F	AAAGTCATTGGACAGGATAGCA	SUMO1-R	TCTCTGACCCTCAAAGAGAAAC
RanGAP1-F	GATCTCACTAGGGGAAGGACTC	RanGAP1-R	CACAGTTGTTGAGCTTGAGTTC
COL I-F	AACGTGGTTTTCTCACCCTAT	COL I-R	CAATCTTGAATCCCATAGCTGC
PAI 1-F	AAAGATGGACTCAACGGTCTC	PAI 1-R	CATCGTGAGCCTTCTCTTGAG
GAPDH-F	GGAGCGAGATCCCTCCAAAAT	GAPDH-R	GGCTGTTGTCATACTTCTCATGG

Small interfering RNAs sequence used for transfection

Gene	Sequence(5' to3')
si-SUMO1-1	GACAGGGUGUUCCAAUGAATT
si-SUMO1-2	GAGAAUUGCUGAUAAUCAUTT
si-SUMO1-3	GGCUUGUGGUGAUAAAUAATT
si-RanGAP1-1	GCCACUGGAGUGACAUGUUTT
si-RanGAP1-2	CCGAGACCUUGAAGACCUUTT
si-RanGAP1-3	GCGUGAAUCCCUACCACUATT
si-CRM1-1	CCAGCAAAGAAUGGCUCAATT
si-CRM1-2	GGAACAUGAUCAACUUAUATT
si-CRM1-3	GGUGGAGAGAGUGAAACAUTT
